# In-stent Carlino—a novel bailout for device-uncrossable in-stent chronic total occlusions

**DOI:** 10.3389/fcvm.2025.1706046

**Published:** 2026-05-12

**Authors:** Ahmad Samir, Hossameldin Hussein

**Affiliations:** 1 Kasr AlAiny Medical School, Faculty of Medicine, Cairo University, Cairo, Egypt; 2 Cardiac Center Hail, Hail, Saudi Arabia; 3 Queen Elizabeth University Hospital, Birmingham, United Kingdom

**Keywords:** Carlino, device uncrossable, hydraulic modification, in-stent chronic total occlusion, microcatheter

## Abstract

**Background and aims:**

In-stent chronic total occlusion (IS-CTO) remains a challenging subset of coronary interventions, frequently demonstrating device uncrossability despite successful wire crossing. The in-stent Carlino technique can serve as a feasible and safe bailout strategy for device-uncrossable IS-CTOs.

**Methods:**

We present two cases of complex IS-CTOs in which successful wire crossing was achieved but device delivery failed despite exhausting appropriate support techniques. In-stent Carlino was performed using controlled contrast injection (0.5 mL) through a wedged microcatheter to create hydraulic plaque modification.

**Results:**

Both cases achieved successful device delivery following in-stent Carlino application. The technique facilitated microcatheter advancement and subsequent lesion treatment without complications. The metallic stent framework and dense neoatherosclerotic tissue provided natural containment, preventing extension of the dissection to extra-plaque.

**Conclusions:**

In-stent Carlino offers an effective bailout for device-uncrossable IS-CTOs. Compared with conventional Carlino applications, safety is enhanced by the natural containment provided by the stent architecture.

## Highlights

- In-stent chronic total occlusions (IS-CTOs) are often characterized by tough, resistant neoatherosclerosis, frequently making device delivery (device uncrossable) and lesion dilation (lesion undilatable) particularly challenging.
- Device uncrossability (when a microcatheter or low-profile balloon cannot cross) occurs more frequent in IS-CTOs compared to native vessel CTOs due to the pathophysiologic foundation.
- The in-stent Carlino technique provides an effective and safe bailout strategy, utilizing hydraulic plaque modification to overcome device uncrossability in IS-CTOs.
- The metallic frame of the failed stent and the dense neoatherosclerotic plaque in IS-CTOs tend to confine the injected contrast intra-plaque, minimizing the risk of subintimal dissection, which often limits success in conventional Carlino applications.

## Background

The Carlino technique, originally developed for CTO recanalization through hydraulic dissection (1), has largely been abandoned due to safety concerns regarding uncontrolled subintimal extension and the development of superior crossing strategies (2).

In-stent chronic total occlusions (IS-CTOs) represent a particularly challenging subset of CTOs, characterized by dense fibrocalcific neoatherosclerosis, frequently resulting in device uncrossability despite successful wire crossing. This phenomenon occurs more frequently and is more likely to be the primary cause of failure in IS-CTO cases compared with native-vessel CTOs (3, 4).

This article presents two illustrative cases demonstrating how device uncrossability in IS-CTOs can be effectively managed using the application of the hydraulic microdissections based on the Carlino technique. However, rather than targeting wire crossing to the distal true lumen, this approach employs controlled hydraulic modification of device-uncrossable in-stent CTO to undermine resistant neoatherosclerotic tissue and facilitate device delivery. Importantly, this technique is likely to have a better safety profile compared to conventional Carlino applications due to the tendency of the pre-existing stent framework and the compact fibrocalcific in-stent neoatherosclerosis to confine the injected contrast intra-plaque.

### Demonstrative Case #1

A 69-year-old man with a medical history of diabetes mellitus (DM), hypertension, chronic obstructive pulmonary disease, and chronic coronary artery disease (CAD) presented with progressive angina despite optimal medical therapy. Coronary angiography (CAG) revealed a long in-stent chronic total occlusion of the right coronary artery (RCA), aged at least 3 years, extending from the para-ostium to the mid-vessel with an estimated length of 35–40 mm. Additional findings included 80% in-stent restenosis (ISR) in the mid-left anterior descending (LAD) artery and 90% stenosis in the proximal left circumflex (LCx) artery. The LAD ISR was treated with sequential non-compliant and drug-coated balloon (DCB) angioplasty, while the LCx stenosis was managed with a single drug-eluting stent (DCB) following optimal lesion preparation.

After 8 weeks, the patient returned for a dedicated percutaneous coronary intervention (PCI) of the RCA in-stent CTO. Anticipating procedural challenges, a 7-French Amplatz Left-1 guide catheter was selected for RCA engagement and adjusted in power position for optimal support. A workhorse wire was placed in the conal branch to further enhance guide stability. Antegrade wire escalation was initiated using a Miracle-12 guidewire delivered through a Corsair Pro microcatheter (MC) (Asahi Intecc Co., Nagoya, Japan).

Despite successful wire crossing through the entire 40-mm IS-CTO segment and confirmed positioning in the distal true lumen via retrograde injections, the Corsair Pro consistently failed to advance beyond the 90° angulation in the proximal-to-mid RCA transition. This resistance was likely secondary to the characteristically dense neoatherosclerosis at the vessel bend. Support was further optimized through anchor balloon inflation in the conal branch and deeper advancement of the Miracle-12 wire into the distal RCA. Nevertheless, repeated torque advancement attempts to the MC failed, with consistent guide disengagement and excessive prolapse of the MC into the aorta.

Failing with the Corsair Pro despite providing all active and passive support maneuvers, a 1.2-mm balloon and then a lower-profile Caravel MC were tried. Both similarly failed to be delivered across the resistant segment.

With conventional approaches exhausted, the MC was positioned at its maximum achievable depth within the IS-CTO body. Following wire removal and confirmation of appropriate positioning, 0.5 mL of contrast was deliberately injected through the MC to perform in-stent Carlino. This controlled hydraulic modification successfully altered the compliance of the densely fibrotic neoatherosclerosis, producing a very satisfying angiographic appearance of cracking the in-stent tissue and visualizing the distal vessel. Following rewiring of the distal true lumen, device delivery and subsequent lesion dilation became feasible.

**Figure 1:**
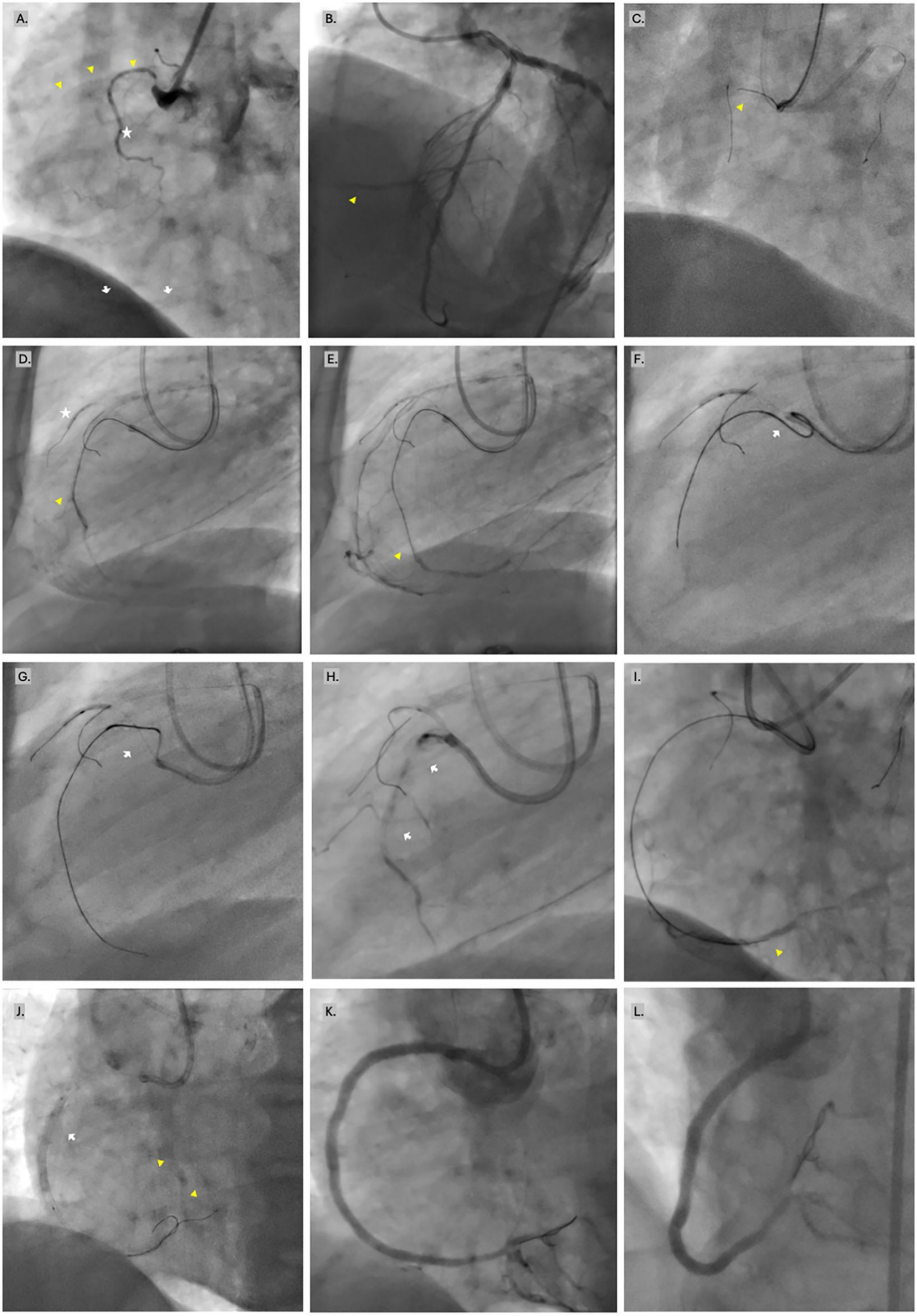
Demonstrative case #1. **(A)** Long RCA IS-CTO starting from the aorto-ostium. The failed stented segment in ostial-to-mid RCA (CTO age: at least 12 months) is marked by the yellow arrowheads. There is a discrete stent in the distal RCA marked by the white arrows. A tiny side branch arises from the RCA para-ostium (star). **(B)** Retrograde filling of the RCA (arrowhead) from the left coronary tree. **(C)** With a 7 French Amplatz left 1 guide and a stabilizing wire in the tiny side branch, antegrade wire escalation started with a Miracle 12 wire (arrow head) supported by a Corsair Pro MC. **(D)** After successful wire crossing of the IS-CTO (arrowhead), advancing the MC was very challenging; hence, support was enhanced by an anchoring balloon in the side branch (star). **(E)** For the inability to cross the lesion by the MC, the Miracle 12 wire was further steered to the distal RCA (arrowhead). **(F,G)** Despite all the maneuvers of active and passive support, attempts to advance the MC persistently lead to guide disengagement and prolapse of the MC (arrow) into the aorta. **(H)** In-stent Carlino performed by wedging the MC tip in the IS-CTO body and injecting 0.5 mL of contrast (arrows). **(I)** Subsequently, the IS-CTO was smoothly wired, and the microcatheter successfully crossed the resistant lesion. **(J)** After predilation, another workhorse wire (arrow heads) was advanced to induce a scoring effect on the neoatherosclerosis with high-pressure inflations of the non-compliant balloon (arrow). **(K,L)** Final results after three DESs.

Due to significant elastic recoil following balloon angioplasty, drug-coated balloon therapy was deemed suboptimal. The lesion was definitively treated with three overlapping DESs, achieving excellent final angiographic results with restoration of coronary TIMI III flow (Figure 1, Supplementary Videos S101–S112).

### Demonstrative Case #2

A 45-year-old man with DM and chronic CAD presented with progressive angina despite optimal medical therapy. His history included a previous PCI to the RCA 10 years earlier and then a PCI to diagonal and obtuse marginal branches 4 years earlier, with CAG at that time revealing an in-stent CTO of the RCA that was managed for conservative therapy. Current CAG at the index presentation revealed a complex “metal jacket” configuration in the RCA, with long-segment stenting from the proximal to distal vessel, proximal subtotal occlusion at the stent inlet, and a long IS-CTO segment extending approximately 45–50 mm to the distal bifurcation.

During the planned PCI session, a 7-French Amplatz Left-1 guide catheter was used for RCA engagement, followed by early angioplasty (3.0 × 18-mm non-compliant balloon at 16 atm) to the proximal subtotal occlusion to relieve pressure dampening and facilitate subsequent device delivery and maneuvering. Antegrade wire escalation was initiated with a Corsair Pro microcatheter and a Gaia 3rd wire (Asahi Intecc Co., Nagoya, Japan). However, despite successful wire crossing into a small distal side branch, gear advancement through the resistant neoatherosclerosis proved extremely challenging, particularly at the angulated RCA segment. Changing to a lower-profile Caravel MC did not solve the impediment. Grenadoplasty with a 1.5 × 20-mm balloon was performed; nevertheless, subsequent attempts to advance the MC failed.

As a last resort, with the microcatheter wedged in a central position within the stented segment, the wire was removed, and in-stent Carlino was performed using 0.5 mL of contrast injection. The resultant hydraulic modification effectively altered tissue compliance, facilitating subsequent MC advancement into the distal true and enabling wire exchange to a Sion wire, which was redirected into the distal posterior descending artery (PDA). Device delivery was successfully achieved and dilation was performed with incrementally sized balloons, facilitated by guide extension support.

Intravascular ultrasound (IVUS) assessment confirmed true lumen positioning throughout the entire intra-stent course. IVUS clearly demonstrated that the microdissections induced by in-stent Carlino were predominantly confined intra-plaque, encaged by the metallic frame of the failed stent and the compact neoatherosclerotic tissue from the extra-plaque (extra-stent) extension.

**Figure 2:**
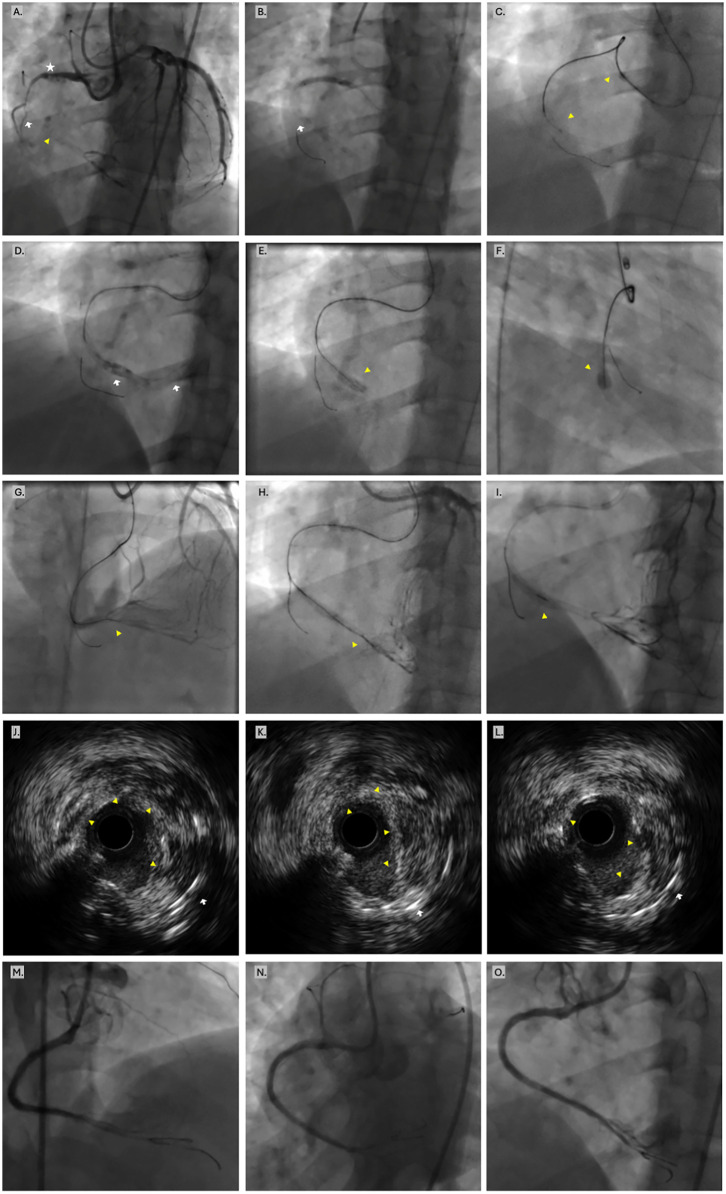
Demonstrative case #2. **(A)** Baseline dual injection revealing proximal RCA 90% ISR (star), followed by a long RCA IS-CTO starting from mid-segment after a marginal branch (arrow). The IS-CTO (yellow arrow head) extends to the distal bifurcation and has estimated age of approximately 18 months. **(B)** After wiring the marginal branch, the proximal ISR was dilated by an appropriately sized non-compliant balloon. **(C)** After the Gaia 3rd wire crossed the IS-CTO, all attempts to torque advance the MC failed with the MC shaft prolapsing into the aorta (arrow heads). **(D)** After wedging the MC at its furthest reach, performed in-stent Carlino (arrows). **(E,F)** Rewiring of the IS-CTO was smooth and confirmed intra-stent course in two projections. **(G,H)** Further advanced the Gaia into the PDA distally, while ensuring true lumen via retrograde injections. **(I)** The MC successfully crossed to the distal true, allowing for exchange to a workhorse wire, then withdrawing the MC (arrowhead). **(J–L)** IVUS imaging demonstrates that the cracks and dissections in the neoatherosclerosis are predominantly contained within the stent frame (intra-plaque). **(M–O)** Final angiographic results after treatment with appropriately sized non-compliant balloons, then two long DCBs.

After adequate preparation as confirmed by IVUS, the IS-CTO was treated with application of two long DCBs (3.0 × 40 mm and 3.5 × 40 mm). Final angiography demonstrated very satisfying results with no complications (Figure 2, Supplementary Videos S201–S208).

## Discussion

The Carlino technique was first introduced decades ago by Professor Mauro Carlino's group as a hydraulic recanalization strategy for coronary chronic total occlusions (1, 5). The original approach involved injecting 3–4 mL of contrast through a microcatheter wedged at the proximal cap to induce hydraulic dissection and facilitate access to the distal true lumen. Over time, the technique evolved toward smaller, more controlled injections (0.5–1.0 mL) in the “mini-Carlino” modification, targeting proximal cap modification and microchannel dilation to enhance wire advancement (6–8).

However, significant advances in dedicated CTO hardware, coupled with improved operator expertise and refined crossing algorithms, have led to the development of more reproducible techniques with superior success rates (9–11). Consequently, the Carlino technique has been largely relegated to a bailout role when conventional strategies fail. This decline was primarily due to concerns regarding potential uncontrolled extension of hydraulic dissection into the subintimal planes, with adverse procedural and long-term outcomes (2, 5, 12). Nevertheless, the concept of utilizing the hydraulic effect of injected contrast to modify the compliance of resistant tissue—targeting “contrast modulation” or “hydraulic recanalization”—survived as a versatile foundation for various applications in CTO practice (8).

IS-CTOs represent a particularly challenging subset of CTO procedures (3, 4), accounting for 5%–15% of all CTO interventions (4, 13). Despite the theoretical advantage of having a defined metallic framework demonstrating the CTO course, IS-CTOs are associated with lower procedural success rates compared to native- vessel CTOs (70%–85% vs. 85%–95%, respectively) (3, 4, 14). This paradox stems from the unique pathophysiological characteristics of in-stent neoatherosclerosis.

The neoatherosclerotic tissue within failed stents is characteristically dense, fibrocalcific, and heavily populated with scattered microcalcifications. This creates a particularly resistant substrate that differs significantly from native atherosclerotic plaque (3). As IS-CTOs age, this neoatherosclerotic tissue becomes increasingly compact and resistant to mechanical disruption (3, 14). Thereby, although device-uncrossable or undilatable lesions are reported in approximately 8.5% of native CTOs, the prevalence can be as high as ≥23% in IS-CTOs (15, 16). Registry data consistently demonstrate that procedural failure despite successful wire crossing—predominantly due to device-uncrossable or undilatable lesions—occurs more frequently in IS-CTOs than in native-vessel CTOs (4, 13).

Moreover, the challenge of device uncrossability is particularly pronounced at anatomical locations with significant vessel tortuosity or sharp angulation, where the combination of dense neoatherosclerosis and geometric constraints creates formidable barriers to device advancement (17). In such scenarios, conventional approaches—including optimal guide catheter support, anchor balloon techniques, guide extensions, and buddy wire strategies—may prove inadequate.

The in-stent Carlino technique addresses device uncrossability through a fundamentally different mechanism while maintaining the core concept of hydraulic plaque modification. Rather than attempting to reach the distal true lumen, this approach specifically targets the resistant neoatherosclerotic tissue of IS-CTOs, which prevents device advancement after successful wire crossing.

The key safety advantage of the in-stent Carlino technique lies in the tendency for natural containment provided by the pre-existing stent architecture. The metallic framework of the failed stent, combined with the dense, fibrocalcific nature of the neoatherosclerotic tissue, creates a protective shield that limits contrast extravasation and subintimal extension. This shielding structure tends to reduce the risk of extensive dissection planes that could compromise procedural success, which was a major caveat of the conventional Carlino.

The hydraulic forces generated by controlled contrast injection create microscopic tissue disruption within the neoatherosclerotic plaque, undermining the substrate and facilitating subsequent device advancement. As demonstrated in the CAG and IVUS images of the presented cases, in-stent Carlino maintains the structural integrity of the overall vessel architecture while locally modifying the compliance of the resistant in-stent atherosclerosis.

Optimal execution of the in-stent Carlino technique requires careful attention to several technical parameters. Contrast volume should be limited to 0.5–1.0 mL per injection, with slow, controlled delivery to allow for gradual tissue modification rather than explosive hydraulic disruption. The MC should be positioned at the point of maximum resistance, typically corresponding to the area where further device advancement fails. This is usually indicated by inability to torque or advance the MC any further in the CTO body, with any additional attempts leading to guide disengagement. The MC tip should be aligned parallel to the IS-CTO segment (not pointing eccentrically) and ideally should be in a central position within the stent frame.

Repeated, step-wise, small-volume injections (<0.5 mL) are preferable to single large-volume injections, allowing for incremental tissue modification while maintaining procedural control. Ultimately, visualizing faint contrast staining into the distal target vessel means adequate achievement of the target tissue breakdown. Ideally, after contrast reaches the distal target with the depicted controlled step-wise injection, rewiring and attempts to progressively advance the MC should be made to assess the effectiveness of plaque modification vs. the need to repeat the technique.

Patient selection should favor cases where conventional support-enhancing techniques (anchor balloon, buddy wire, guide extension) have been exhausted and proven inadequate, and where the risk–benefit ratio supports additional intervention attempts. Unfavorable scenarios may include situations where extensive dissection already exists or where the risk of vessel perforation is deemed high.

Dual-lumen microcatheters (DLMCs) represent a potential technical refinement that could enhance the safety profile of the in-stent Carlino technique (18). The theoretical advantage lies in maintaining wire position within the distal true lumen during contrast injection, thereby eliminating the need for rewiring and reducing the risk of losing distal access—a critical consideration in complex IS-CTO interventions. However, the majority of currently available DLMC platforms typically feature larger crossing profiles and reduced torque transmission compared with single-lumen microcatheters. These characteristics may compromise their crossability and deliverability to achieve optimal progression within resistant, device-uncrossable IS-CTO segments, which is fundamental to the technique's success. The reduced pushability and trackability of DLMCs could paradoxically limit utility in the precise clinical situations (resistant, device-uncrossable IS-CTO) where in-stent Carlino is most needed as a bailout strategy.

Neither of the presented cases involved evaluation of DLMCs, as single-lumen microcatheters were successfully advanced to the point of maximal resistance. The comparative performance of DLMCs remains to be evaluated in appropriately selected cases, particularly in scenarios where the trade-off between enhanced safety and reduced deliverability may be justified by lesion complexity or operator preference.

Several alternative approaches exist for managing device-uncrossable IS-CTOs, each with distinct advantages and limitations (19). Rotational atherectomy has been employed for heavily calcified IS-CTOs but carries risks of vessel perforation and may be challenging in tortuous anatomies. Another major limitation for rotational atherectomy in device-uncrossable lesions is the need to deliver the rotawire to the distal true lumen, which traditionally necessitates crossing the in-stent CTO with an MC. This limitation makes rotational atherectomy less suited for overcoming device uncrossability, though it still remains an excellent bailout for undilatable lesions. On the other hand, laser atherectomy has the advantage of applicability over any 0.014 in. wire and can offer precise and effective tissue modification. Nevertheless, requiring specialized equipment and expertise that are not universally available may be the main limitation for its use. Balloon lithotripsy represents an emerging technology for modifying calcified plaques, though owing to its large, unfriendly profile, application in a CTO that cannot be crossed by low-profile balloons or MC is not impractical as a bailout strategy. Super high-pressure (OPN non-compliant) balloons and cutting/scoring balloons have proven effective in modifying the resistant neoatherosclerotic tissue in stent failure; however, their large profiles could limit their utility (20, 21). Similar to rotational atherectomy and balloon lithotripsy, these specialty balloons may better serve for undilatable lesions, but are less likely to overcome device uncrossability.

In summary, in-stent Carlino probably can improve overall IS-CTO procedural success rates by providing a readily available bailout option using standard, low-cost catheterization laboratory equipment, potentially reducing the need for staged procedures or surgical revascularization. These two illustrative cases demonstrate that the in-stent Carlino technique can serve as an effective bailout strategy when conventional approaches to device uncrossability have been exhausted. The technique's reliance on standard catheterization laboratory equipment and familiar procedural concepts enhances its practical applicability across diverse clinical settings. The improved safety profile—stemming from the tendency to confine injected contrast intra-plaque and reduce the risk of uncontrolled dissection—represents a significant advantage over conventional Carlino applications. Owing to its expected effectiveness, practicality, and safety, the in-stent Carlino technique can provide a valuable addition to the armamentarium for managing complex IS-CTOs. The presented cases provide a foundational concept for the technique and highlight its clinical utility as an innovative approach to overcome device uncrossability in IS-CTOs.

### Limitations and considerations

Several limitations must be acknowledged regarding the in-stent Carlino technique. The two presented cases illustrate early experience with a limited long-term follow-up data. Nevertheless, together with the provided rationalization for the technique, they provide the conceptual foundation and technical methodology for utilizing in-stent Carlino in such a frequently encountered challenge. Optimal patient selection criteria require further refinement through wider-scale evaluation. The technique may not be effective in all anatomical configurations or plaque morphologies, underscoring the critical need for appropriate case and anatomy selection. Moreover, the learning curve for operators may vary, as careful attention to step-wise controlled injection of ultra-small contrast volumes is essential for safe implementation. Furthermore, while the theoretical safety advantages are compelling, comprehensive safety data from larger patient cohorts are needed to fully establish the risk profile of this approach.

### Future directions

The in-stent Carlino technique represents an evolution in CTO intervention strategies, demonstrating how established concepts can be repurposed for novel applications. In-stent Carlino offers the advantage of utilizing standard catheterization laboratory equipment and techniques familiar to most interventional cardiologists. Future efforts should focus on defining the appropriate patient and anatomy selection, establishing standardized technical protocols, and conducting comparative studies with alternative bailout strategies.

Long-term angiographic and clinical outcomes data are needed to fully establish the role of this technique in contemporary CTO practice. In addition, the role of adjunctive technologies, such as intravascular imaging guidance, should be investigated to further optimize technique performance and safety.

The cases presented demonstrate that the in-stent Carlino technique offers a valuable addition to the armamentarium for managing challenging IS-CTOs, providing an effective bailout solution for device uncrossability while maintaining an improved safety profile compared with conventional Carlino applications.

## Conclusions

The in-stent Carlino technique represents a paradigmatic evolution in the management of device-uncrossable in-stent CTO. By repurposing the established concept of hydraulic plaque modification, this approach addresses a significant limitation in contemporary CTO intervention practice. The unique pathophysiological characteristics of in-stent neoatherosclerosis, while creating resistant barriers to device advancement, simultaneously provide a structural foundation for safe and controlled hydraulic modification. The metallic framework of the failed stent, combined with the dense fibrocalcific nature of the neoatherosclerotic tissue, creates a natural containment system that can reduce the risk of extra-plaque extension compared with conventional Carlino applications. Further investigation, larger clinical series, and long-term outcomes are needed to fully refine appropriate case selection and establish the role of this technique in contemporary CTO practice.

## Data Availability

The raw data supporting the conclusions of this article will be made available by the authors without undue reservation.

## Supplementary material

Supplementary File 1 (case 1) Videos Video 101 → LAO caudal for LCA baseline. Video 102 → AP cranial for LCA baseline. Video 103 → RCA baseline. Video 104 → LAO caudal following PCI to LAD-LCx. Video 105 → AP cranial following PCI to LAD-LCx. Video 106 → Baseline dual injection for PCI to RCA CTO. Video 107 → Device uncrossable 01. Video 108 → Device uncrossable 02. Video 109 → IS-Carlino to IS-CTO. Video 110 → MC delivered distal RCA. Video 111 → Final LAO cranial. Video 112 → Final AP cranial.

Supplementary File 2 (case 2) Videos Video 201 → Baseline dual injection. Video 202 → Dilating severe proximal RCA ISR. Video 203 → Device uncrossable. Video 204 → IS-Carlino. Video 205 → Smooth rewiring. Video 206 → Advanced MC then exchanging to workhorse wire. Video 207 → Guideliner—lesion preparation—antegrade injection. video 208 → è DCB and final results
